# Intraoperative malignant glaucoma during femtosecond laser-assisted cataract surgery: A case report

**DOI:** 10.1097/MD.0000000000029250

**Published:** 2022-06-24

**Authors:** Rong Xu, Danmin Cao, Ya Jiao, Qingyan Zeng

**Affiliations:** Aier Eye Hospital of Wuhan University, Wuchang District, Wuhan, China.

**Keywords:** case report, femtosecond laser-assisted cataract surgery, malignant glaucoma

## Abstract

**Rationale:**

Femtosecond laser-assisted cataract surgery (FLACS) has grown in popularity among ophthalmologists as a novel surgical technique. However, malignant glaucoma (MG) is a complication of FLACS. Herein, we report a case of MG following FLACS.

**Patient concerns:**

A 66-year-old woman presented with complaints of blurred vision in the right eye and a foreign body sensation in both eyes. Ophthalmological examinations showed that the corrected distance visual acuity was 20/50 and 20/25 in the right and left eyes, respectively. Without any topical anti-glaucoma medication, the intraocular pressure (IOP) was 20 mmHg in the right eye and 17 mmHg in the left eye. Slit-lamp examination of the right eye revealed a transparent cornea with a defect in the punctate overlying epithelium; the central anterior chamber depth was shallow the peripheral iris laser shot was visible, the pupil was normal, and the lens was mainly cortical opacified.

**Diagnoses:**

Based on the patient's symptoms, examination results, and preliminary diagnoses, age-related cataract in the right eye, binocular post-antiglaucoma surgery, pseudophakicin in the left eye, and Sjogren syndrome were included.

**Interventions:**

FLACS was performed to facilitate anterior capsulotomy and segmentation of the nucleus in the right eye. MG occurred after the femtosecond procedure, and with the treatment of medicines combined with phacoemulsification, IOP was eventually normal without further antiglaucoma therapy.

**Outcomes:**

IOP was 16 mmHg on postoperative day 1. Ocular ultrasonography revealed no choroid detachment or hemorrhage in the right eye. Two weeks postoperatively, uncorrected visual acuity was 20/25, and IOP remained normal with no further antiglaucoma treatment on 1 month postoperatively.

**Conclusions:**

We describe the occurrence of MG after FLACS and illustrate that miosis and bubble formation after FLACS may be risk factors for MG during FLACS.

## 1 Introduction

Malignant glaucoma (MG) is a rare but serious complication, which was first reported by von Graefe in 1869.^[1]^ It is characterized by a shallow or disappeared anterior chamber (AC) with markedly elevated intraocular pressure (IOP); however, IOP can be normal in the early stages in some patients.^[2]^ The incidence of MG is approximately 1.45% among all phacoemulsification procedures and 0.6% to 4% in primary angle-closure glaucoma surgery.^[2,3]^ However, MG complications during femtosecond laser-assisted cataract surgery (FLACS) are extremely rare. To further study and make better use of the FLACS technique in the future, we report a case of MG following FLACS in which the patient presented with very high IOP and the AC was unexpectedly shallow.

## 2 Case presentation

This retrospective case report was conducted with the approval of the Institutional Review Board of Wuhan Aier Eye Hospital and adhered to the tenets of the Declaration of Helsinki. A 66-year-old woman presented with gradual and painless decreasing vision without obvious inducement in the right eye for 2 years, which was frequently accompanied by a foreign body sensation with eye redness. Seven years prior, the patient underwent phacoemulsification combined with trabeculectomy in the left eye for acute angle-closure glaucoma. The fellow eye was prophylactically treated with neodymium:yttrium-aluminum-garnet laser peripheral iridotomy. The patient was diagnosed with Sjögren syndrome 5 years previously.

**Figure 1:**
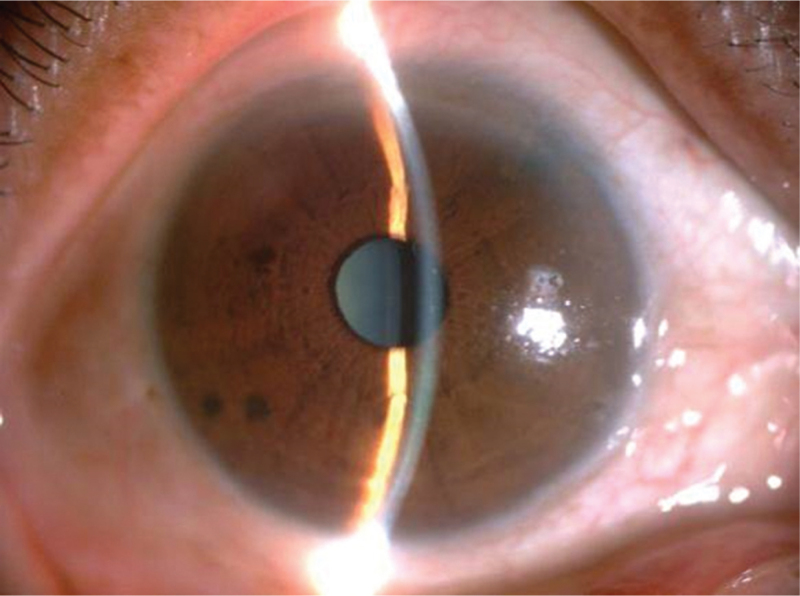
Preoperative anterior segment image showing shallow anterior chamber in the right eye.

Ophthalmological examinations showed that the corrected distance visual acuity was 20/50 and 20/25 in the right and left eyes, respectively. Without any topical antiglaucoma medications, the IOP was 20 mmHg in the right eye and 17 mmHg in the left eye. Slit-lamp examinations of the right eye (Fig. 1) revealed a transparent cornea with a defect in the punctate overlying epithelium; the central anterior chamber depth (ACD) was almost equivalent to 2 corneal thicknesses (CT); the peripheral ACD was less than one-fourth of the CT, the peripheral iris laser shot was visible, and the pupil was normal; the lens was mainly cortical opacified, which was a grade 3 nuclear cataract (nuclear opalescence, 3; nuclear color, 3; according to the Lens Opacities Classification System III).^[4]^ The right eye ocular parameters, as measured by a Tomey OA-2000 biometer, of axial length, mean corneal curvature, CT, ACD, and lens thickness were 22.57 mm, 44.28 D, 585 μm, 1.96 mm, and 5.05 mm, respectively. Ocular fundus examination of the right eye was unremarkable, with a cup-to-disc ratio of 0.3, a healthy neuroretinal rim, and a retina without hemorrhage. Narrow AC angles in all quadrants of the right eye were noted on ultrasound biomicroscopy. Binocular visual field examination revealed no obvious abnormalities. Based on these features, we considered that the preliminary diagnoses included age-related cataract in the right eye, binocular post-antiglaucoma surgery, pseudophakicin in the left eye, and Sjögren syndrome.

**Figure 2:**
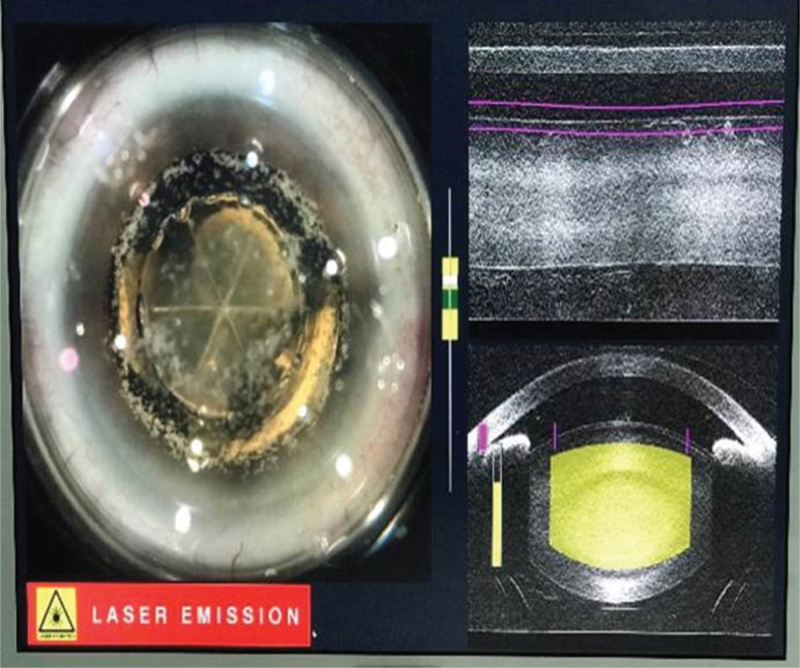
Optical coherence tomography image showing a successful capsulotomy and nucleus divisions.

**Figure 3:**
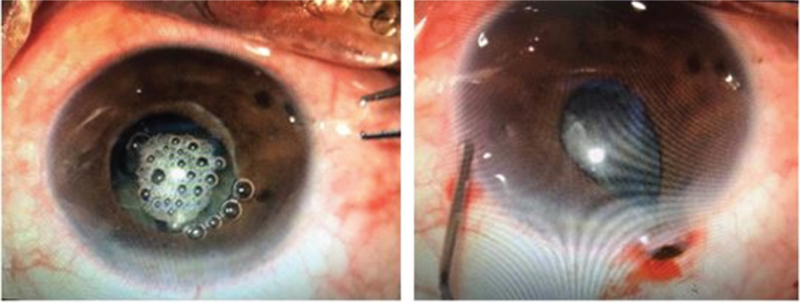
Intraoperative image of malignant glaucoma with axial shallow anterior chamber, progressive contraction of pupil, and prolapsed iris.

**Figure 4:**
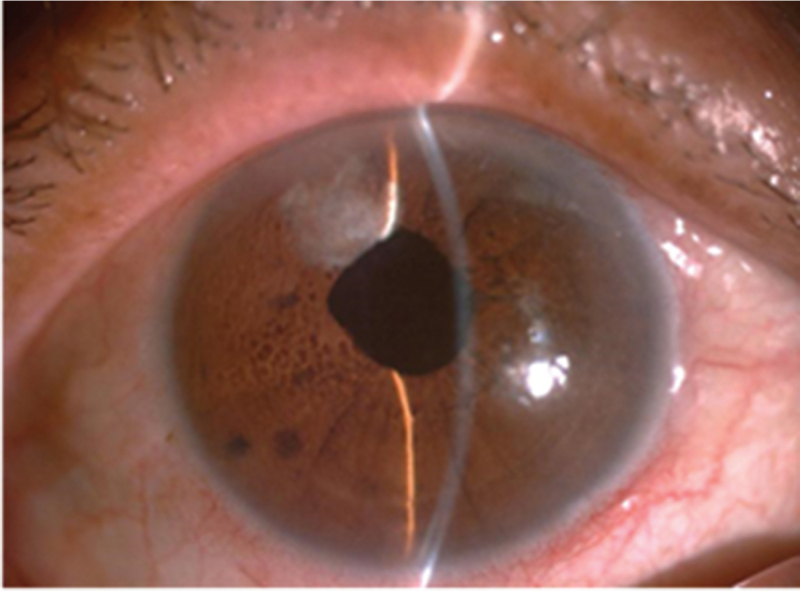
Postoperative anterior segment image showing normal anterior chamber.

Chondroitin sulfate, tacrolimus, and ofloxacin eye ointments were used to relieve dry eye symptoms (2 weeks before the operation), and the patient gave consent to undergo FLACS. Considering the poor ocular surface, no preoperative nonsteroidal anti-inflammatory drugs were administered. To avoid precipitating acute angle closure, 20% (w/v) intravenous mannitol was applied 30 minutes before surgery. The femtosecond laser procedure was successfully performed using the LenSx Laser System version 2.3 (Alcon Laboratories Inc., Fort Worth, TX) (Fig. 2), after patient interface docking, a 5.2 mm diameter capsulotomy was created with 6-mJ laser energy. Six pieces in a cross pattern were used for lens fragmentation with 12-mJ laser energy. After completion of the laser procedure, the patient was returned to the operating microscope. Unexpectedly, an extremely shallow AC and progressive contraction of the pupil were noted (Figs. 3 and 4). The IOP was markedly increased on digital tonometry. Relevant measures were immediately implemented, including massaging the eyeball, releasing the aqueous humor from the auxiliary incision, and injecting 0.1% adrenaline from the primary incision to assist mydriasis. The iris suddenly prolapsed, the pupil further shrunk to 2 mm in diameter, and IOP was accurately measured to be 69 mmHg using an l-CARE rebound tonometer. We initially considered that this indicated MG rather than suprachoroidal expulsive hemorrhage, which compelled us to suspend the surgery. The patient was then started on 1% atropine ointment every 30 minutes; compound tropicamide, 15 minutes per time, for 4 consecutive times; and other topical IOP-lowering medications (timolol maleate 0.5% once and 0.15% brimonidine once). The IOP gradually decreased to 38 mmHg 2 hours later, and the pupil diameter returned to 5 mm. Phacoemulsification was then performed. After removing the lens nucleus, the IOP further decreased. The IOP was 16 mm Hg on postoperative day 1. Ocular ultrasonography revealed no choroid detachment or hemorrhage in the right eye. Two weeks postoperatively, uncorrected visual acuity was 20/25, IOP remained normal with no further antiglaucoma treatment, and a uniformly deep AC was evident (Fig. 4). The loss rate of endothelial cells was 13.86%. Both the shape of the optic disc and the thickness of the retinal nerve fiber layer by optical coherence tomography were normal, and there was no abnormality in the visual field examination. The duration of follow-up was 1 month, IOP remained normal in the right eye.

## 3 Discussion

MG is a rare but severe complication of anterior segment surgery; however, its pathophysiology is poorly understood. The abnormal anatomic relationships between **ciliary body processes, lens, and anterior vitreous** appear to contribute to the development of MG, which leads to aqueous misdirection into the posterior segment.^[5,6]^ Generally, MG is described following ophthalmic procedures, such as various anti-glaucoma operations, cataract surgery,^[7,8]^ corneal transplantation, vitrectomy, and cyclophotocoagulation. MG has also been reported in the literature after laser iridotomy,^[5]^ posterior capsulotomy,^[9]^ and phakic posterior chamber intraocular lens implantation.^[10,11]^ However, to the best of our knowledge, complications of MG that have occurred during FLACS are extremely rare, and FLACS is rapidly growing in popularity owing to its unique accuracy, predictability, and safety.^[12]^ However, MG complications of MG are extremely rare. Only Grewal and Basti reported a unique MG that occurred in a patient with phacomorphic angle closure during FLACS.^[13]^

Abnormal anatomical structures are recognized risk factors for developing MG, including a small cornea, shallow AC, short ocular axis, thick anterior lens, and small ciliary ring. Angle-closure glaucoma, hyperopia, nanophthalmos, and plateau iris configuration are predisposed to MG.^[14]^ Undoubtedly, the anatomical abnormalities of the extremely shallow AC, short axial length, intumescent cataract, and narrow AC angle in our patient increased the risk for MG. Femtosecond laser surgery may have primarily contributed to the occurrence of MG. In our opinion, there is a sequence of potential pathomechanisms for MG related to femtosecond laser surgery. First, suction during femtosecond laser pretreatment could also increase IOP, leading to forward lens movement and AC flattening, axially and peripherally. Second, no preoperative nonsteroidal anti-inflammatory drugs were administered because of the poor ocular surface. Intraoperative miosis that resulted from the significantly higher levels of prostaglandins and inflammatory cytokines combined with the formation of intralenticular gas after femtosecond laser pretreatment may have played a major role in the MG complication in this high-risk patient.^[15]^ The constriction of the pupil broke the aqueous outflowing egress and precipitated the forward movement of the lens, which subsequently resulted in pupillary block through a vicious cycle. This mechanism may be consistent with that observed in patients who have undergone laser peripheral iridotomy.^[16]^ Furthermore, the presence of laser fragmentation of the lens combined with bubbles within both the AC and lens could increase the intracapsular volume, which may ultimately lead to the formation of the iris–lens diaphragm.

## 4 Conclusion

This case emphasizes the potential for MG complication during FLACS, especially in patients at a high risk for MG. Careful preoperative evaluation and preparation is important, as MG can be successfully managed with appropriate and timely interventions.

## Author contributions

XR performed the surgery, analyzed the data, and obtained funding; CDM acquired data, drafted the article, and obtained funding; JY acquired data; and ZQY conceived the study. All authors reviewed the manuscript, and read and approved the final manuscript.

**Conceptualization:** Qingyan Zeng, Rong Xu

**Data curation:** Danmin Cao, Rong Xu, Ya Jiao

**Funding acquisition:** Danmin Cao, Rong Xu

**Investigation:** Rong Xu, Ya Jiao

**Methodology:** Danmin Cao, Qingyan Zeng, Rong Xu

**Project administration:** Rong Xu

**Supervision:** Danmin Cao, Qingyan Zeng, Ya Jiao

**Validation:** Qingyan Zeng

**Visualization:** Danmin Cao, Qingyan Zeng

**Writing – original draft:** Danmin Cao, Rong Xu, Ya Jiao

**Writing – review & editing:** Danmin Cao, Qingyan Zeng, Rong Xu, Ya Jiao
